# Diclofenac-Induced Cytotoxicity in Direct and Indirect Co-Culture of HepG2 Cells with Differentiated THP-1 Cells

**DOI:** 10.3390/ijms23158660

**Published:** 2022-08-04

**Authors:** Atsushi Kawase, Ouka Takashima, Satsuki Tanaka, Hiroaki Shimada, Masahiro Iwaki

**Affiliations:** 1 Department of Pharmacy, Faculty of Pharmacy, Kindai University, 3-4-1 Kowakae, Higashiosaka 577-8502, Osaka, Japan; 2 Pharmaceutical Research and Technology Institute, Kindai University, 3-4-1 Kowakae, Higashiosaka 577-8502, Osaka, Japan; 3 Antiaging Center, Kindai University, 3-4-1 Kowakae, Higashiosaka 577-8502, Osaka, Japan

**Keywords:** NSAIDs, macrophages, co-culture, liver, cytotoxicity

## Abstract

Non-steroidal anti-inflammatory drugs (NSAIDs) such as diclofenac (DIC) frequently induce drug-induced liver injury (DILI). It is unclear whether macrophages such as M1 and M2 participate in NSAID-associated DILI; elucidating this relationship could lead to a better understanding of the detailed mechanism of DILI. We co-cultured human hepatoma HepG2 cells with M1 or M2 derived from human monocytic leukemia THP-1 cells to examine the roles of M1 and M2 in DIC-induced cytotoxicity. DIC was added to the direct or indirect co-cultures of HepG2 cells with M1 or M2 (HepG2/M1 or HepG2/M2, respectively) at cell ratios of (1:0, 1:0.1, 1:0.4, and 1:1). In both direct and indirect HepG2/M2 co-cultures (1:0.4), there was lower lactate dehydrogenase release compared with HepG2/M1 co-cultures. Other NSAIDs as well as DIC showed similar protective effects of DIC-induced cytotoxicity. There were only slight differences in mRNA levels of apoptosis- and endoplasmic reticulum stress-associated factors between M1 and M2 after DIC treatment, suggesting that other factors determined the protective effects of M2 on DIC-induced cytotoxicity. Levels of high mobility group box 1 (HMGB1) in the medium and the mRNA expression levels of HMGB1 receptors were different between M1 and M2 after DIC treatment. Increased HMGB1 concentrations and expression of toll-like receptor 2 mRNA in M1 were observed compared with M2 after DIC treatment. In conclusion, these results suggested that the HMGB1/TLR2 signaling axis can be suppressed in M2 but not M1, leading to the different roles of M1 and M2 in NSAID-induced cytotoxicity.

## 1. Introduction

Drug-induced liver injury (DILI) is a frequent cause of liver injury after treatment with agents such as non-steroidal anti-inflammatory drugs (NSAIDs). Reactive metabolites of NSAIDs (e.g., NSAIDs acyl glucuronide (NSAIDs-AG) and benzoquinone imine) and/or macrophages are involved in DILI [1,2,3,4]. NSAIDs-AG is formed from NSAIDs catalyzed by UDP-glucuronosyltransferases (UGTs) such as UGT1A9 and UGT2B7 and is excreted into the bile via multidrug resistance-associated protein 2 (MRP2). Our group previously showed the involvement of NSAIDs-AG in inducing DILI [5] and established an in vitro system to evaluate DILI using isolated murine hepatocytes co-cultured with peritoneal macrophages [6]. Another group demonstrated that a test using monocyte-derived hepatocyte-like cells was useful to identify DILI-associated drugs [7]. Macrophages are generally classified into M1 (a classical macrophage activation) and M2 (alternative macrophage activation). It is unclear whether M1 and/or M2 participate in NSAID-associated DILI. Elucidating the role of M1 and M2 in DILI induction is important to understand the detailed mechanism of NSAID-associated DILI and to develop preventive strategies for severe DILI. Danan and Teschke demonstrated that the main cause of DILI induction was treatment of NSAIDs in the Hawaii Department of Health with the application of RUCAM in a heterogeneous group of patients after use of multiple dietary supplements and synthetic drugs. Therefore, it is important to clarify the regulation of NSAIDs-induced DILI [8].

Diclofenac (DIC) is an NSAID used to treat patients with pain, fever, and inflammation that is metabolized to DIC-AG by UGT2B7 and occasionally induces severe DILI [9,10,11,12,13]. DIC-AG forms covalent adducts with endogenous proteins, potentially changing protein function or inducing immune responses [14,15]. It is generally assumed that DIC-induced cytotoxicity is caused by DIC itself and reactive metabolites of DIC. However, it remains unclear whether M1 and M2 are involved in DIC-induced cytotoxicity. In addition to DIC, we also tested other NSAIDs including flurbiprofen (FLU), ibufenac (IBF), ibuprofen (IBP), ketoprofen (KET), naproxen (NAP), and zomepirac (ZOM).

To examine the role of M1 and M2 in NSAID-associated DILI, we used human hepatoma HepG2 cells co-cultured with M1 or M2 derived from phorbol 12-myristate 13-acetate (PMA)-differentiated human monocytic leukemia THP-1 cells (M0). M1 and M2 were differentiated from M0 using interferon (IFN)-γ and lipopolysaccharide (LPS), and interleukin (IL)-4 and IL-13, respectively [16]. The ratios of HepG2 cells to M0, M1, or M2 (HepG2/M0, HepG2/M1, or HepG2/M2) were set at 1:0, 1:0.1, 1:0.4, and 1:1, because the 1:0.1 and 1:0.4 ratio reflected the physiological and inflamed condition [17], respectively, and 1:0 and 1:1 were a condition of lower and higher ratios of macrophages compared with the physiological condition to clarify the roles of macrophages in DIC-induced cytotoxicity. We also performed direct and indirect co-culture of HepG2 with M0, M1, and M2 to clarify whether either humoral factors or intercellular contacts were needed for the regulation of DIC-induced cytotoxicity. To clarify the involvement of high mobility group box 1 (HMGB1) in DIC-induced cytotoxicity, we measured the HMGB1 concentrations in the medium and the mRNA levels of HMGB1 receptors such as toll-like receptor (TLR)2, TLR4, and the receptor for advanced glycation end products (RAGE). HMGB1 is a nonhistone chromatin-associated protein that plays a key role in the onset of hematopoietic malignancy [18].

## 2. Results

### 2.1. Lactate Dehydrogenase Release from Monocultures of HepG2 Cells, M0, M1, and M2

**Figure 1:**
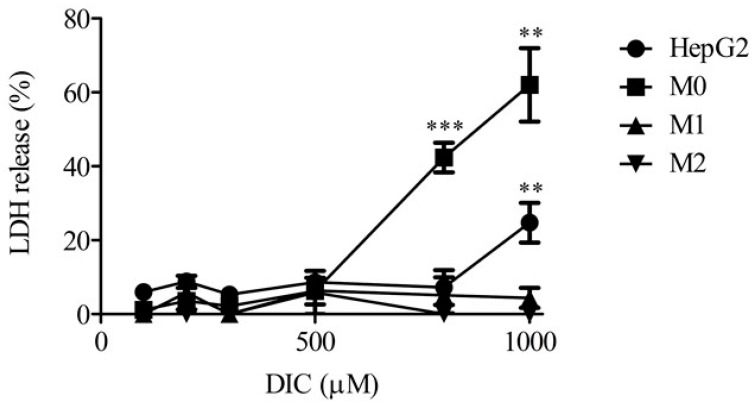
LDH release in HepG2, M0, M1, or M2 alone 24 h after DIC treatment (100, 200, 300, 500, 800, and 1000 μM). The results are expressed as the mean ± SE of each group (n = 3–6). Significant differences (** *p* < 0.01 and *** *p* < 0.001 vs. DIC (100 μM)) were observed.

Figure 1 shows levels of lactate dehydrogenase (LDH) release into the medium from monocultures of HepG2 cells, M0, M1, and M2 24 h after DIC treatment. Relatively low levels of LDH release were detected in all groups with up to 500 µM DIC. In HepG2 cells treated with 1000 µM DIC, and in M0 treated with 800 or 1000 µM DIC, significantly higher levels of LDH were observed compared with 100 µM DIC treatment. Therefore, we chose a dose of 500 µM DIC in the following experiments because little cytotoxicity occurred in all groups at 500 µM DIC.

### 2.2. LDH Release in Direct and Indirect Co-Cultures of HepG2 Cells with M0, M1, and M2

**Figure 2:**
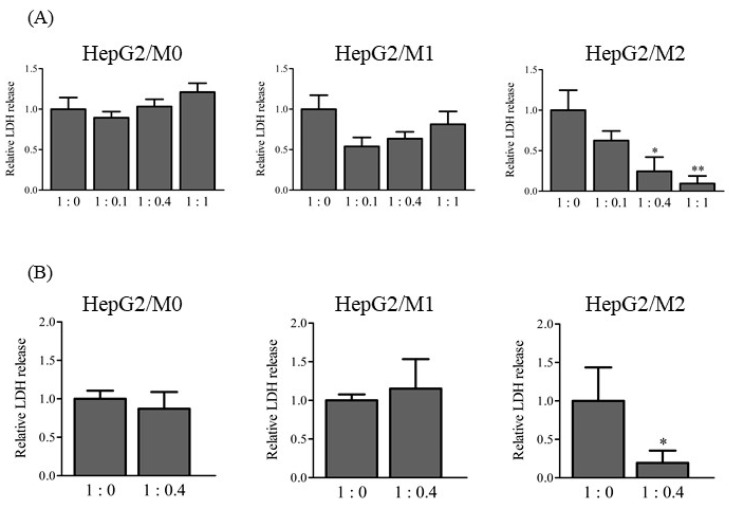
Relative LDH release to 1:0 in direct co-culture HepG2/M0, M1, and M2 (1:0, 1:0.1, 1:0.4, and 1:1) (**A**), and in indirect co-culture HepG2/M0, M1, and M2 (1:0.4) (**B**) 24 h after DIC treatment (500 μM). The results are expressed as the mean ± SE of each group (n = 3–4). Significant differences (* *p* < 0.05 and ** *p* < 0.01 vs. HepG2/M2 (1:0)) were observed.

To clarify the roles of M1 and M2 in the DIC-induced cytotoxicity of HepG2 cells, LDH release into the medium was measured from direct and indirect co-cultures of HepG2 cells with M0, M1, or M2 (1:0, 1:0.1, 1:0.4, and 1:1) 24 h after DIC treatment (500 µM) (Figure 2). LDH release was similar among all cell ratios in the direct co-culture of HepG2 cells with M0 and M1 (Figure 2A). In contrast, LDH release was significantly decreased in direct co-cultures of HepG2 cells with M2 according to the ratio of M2 to HepG2 cells. Indirect co-culture of HepG2 with M2 but not M0 or M1 (1:0.4) showed a significantly lower LDH release compared with HepG2 cells alone (Figure 2B). The lower LDH release in HepG2/M2 (1:0.4 and 1:1) does not have a physiological meaning.

**Figure 3:**
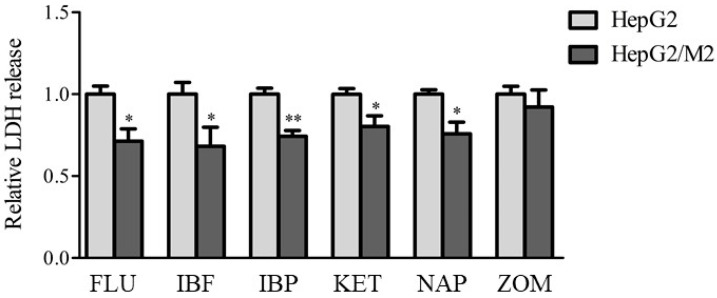
Relative LDH release to HepG2 alone in indirect co-culture HepG2/M2 (1:0.4) 24 h after treatments of FLU (500 μM), IBF (1 mM), IBP (1 mM), KET (1 mM), NAP (1 mM), or ZOM (1 mM). The results are expressed as the mean ± SE of each group (n = 4–5). Significant differences (* *p* < 0.05 and ** *p* < 0.01 vs. HepG2) were observed.

To examine whether similar effects of the presence of M2 with HepG2 cells on cytotoxicity were observed with other NSAIDs, LDH release from HepG2 cells was measured 24 h after treatment with FLU, IBF, IBP, KET, NAP, and ZOM (Figure 3). Co-culture of HepG2 cells with M2 led to decreased LDH release following treatment with FLU, IBF, IBP, KET, and NAP, but not following ZOM.

### 2.3. mRNA Levels of Apoptosis- and ER Stress-Associated Factors and Glutathione Levels

**Figure 4:**
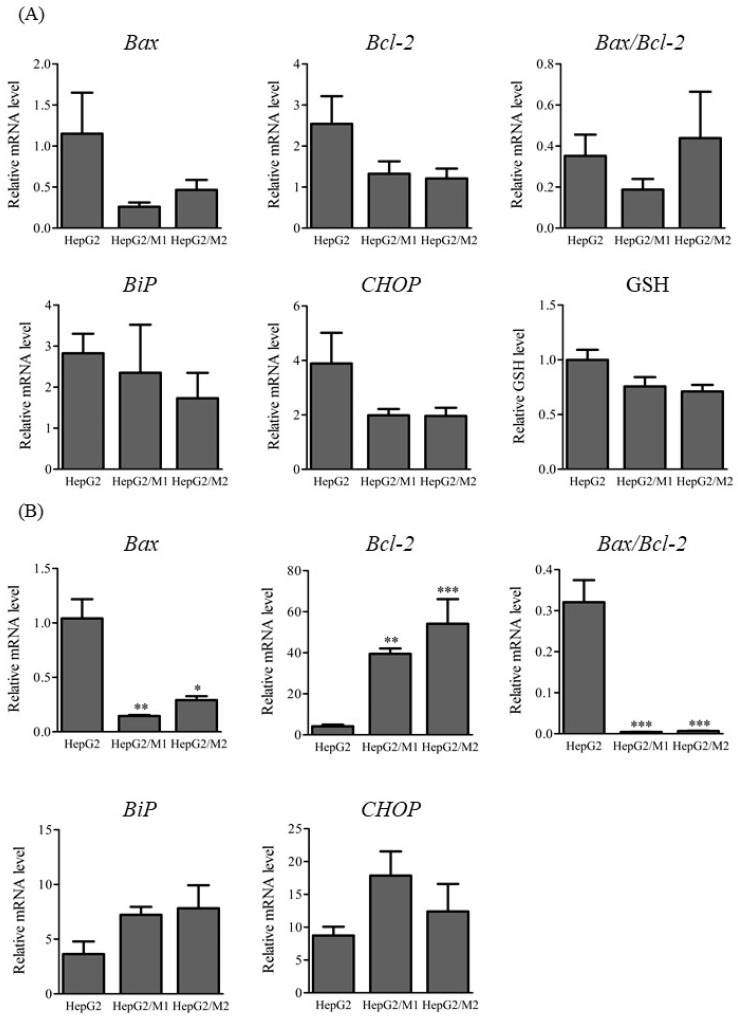
Relative mRNA levels of *Bax*, *Bcl-2*, and the ratios of *Bax* to *Bcl-2* (**A**), and *Bip*, and *CHOP*, and relative GSH levels (**B**) in HepG2, HepG2/M1, and HepG2/M2 3 h and 24 h after DIC treatment (500 μM). The results are expressed as the mean ± SE of each group (n = 4–5). Significant differences (* *p* < 0.05, ** *p* < 0.01, and *** *p* < 0.001 vs. HepG2) were observed.

mRNA levels of apoptosis-associated factors such as the pro-apoptotic genes *Bcl-2-ssociated protein X* (*Bax*) and *B cell lymphoma/leukemia 2* (*Bcl-2*) and endoplasmic reticulum (ER) stress-associated factors such as *Bip* and *CHOP* were measured in HepG2 cells 3 h and 24 h after DIC treatment (500 µM) as well as in HepG2/M1 and HepG2/M2 co-cultures (Figure 4). The relative levels of *Bax* mRNA were significantly decreased when HepG2 cells were co-cultured with M1 or M2. On the contrary, the relative levels of *Bcl-2* mRNA were significantly increased when HepG2 cells were co-cultured with M1 or M2 (Figure 4A). The ratios of *Bax* to *Bcl-2* were markedly decreased 24 h but not 3 h after DIC treatment. The presence of M1 or M2 with HepG2 cells had little impact on *Bax*, *Bcl-2*, *Bip*, or *CHOP* mRNA levels 3 h after DIC treatment (Figure 4B). Intracellular glutathione (GSH) levels were unchanged 3 h after DIC treatment (Figure 4B).

### 2.4. mRNA Levels of UGTs and Transporters

**Figure 5:**
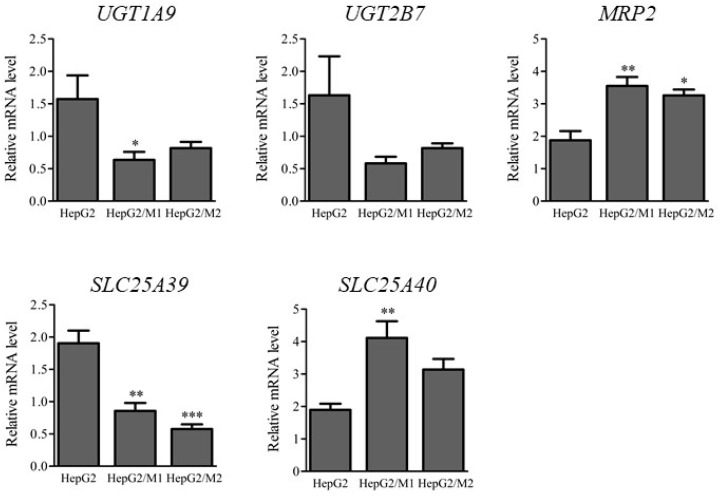
Relative mRNA levels of *UGT1A9*, *UGT2B7*, *MRP2*, *SLC25A39*, and *SLC25A40* in HepG2 24 h after DIC treatment (500 μM) to HepG2, HepG2/M1, and HepG2/M2. The results are expressed as the mean ± SE of each group (n = 4–5). Significant differences (* *p* < 0.05, ** *p* < 0.01, and *** *p* < 0.001 vs. HepG2) were observed.

The mRNA levels of *UGTs* and transporters such as *MRP2*, *SLC25A39*, and *SLC25A40* were determined 24 h after DIC treatment (500 µM) in HepG2 cell monocultures and in HepG2/M1 and HepG2/M2 co-cultures (Figure 5). HepG2/M1 co-cultures showed decreased *UGT1A9* and *SLC25A39* expression and increased *MRP2* and *SLC25A40* levels compared with HepG2 monocultures. HepG2/M2 co-cultures had decreased *SLC25A39* expression and increased *MRP2* levels.

### 2.5. HMGB1 Concentrations in Medium and mRNA Levels of HMGB1 Receptors in M1 and M2

**Figure 6:**
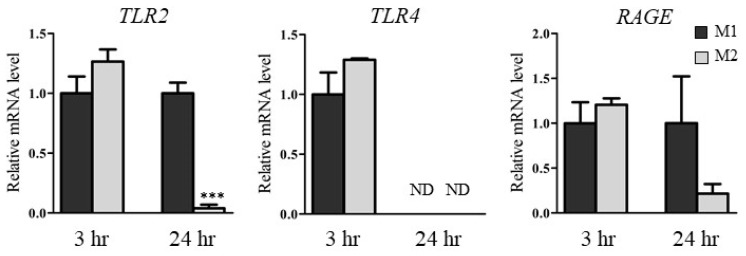
Relative mRNA levels of *TLR2*, *TLR4*, and *RAGE* in M1 and M2 3 h or 24 h after DIC treatment (500 μM) to HepG2, HepG2/M1, and HepG2/M2. The results are expressed as the mean ± SE of each group (n = 3–4). ND indicates not detected. Significant difference (*** *p* < 0.001 vs. M1 of 24 h) was observed.

To clarify the differences in expression of HMGB1 and HMGB1 receptors in M1 and M2, we examined HMGB1 concentrations in conditioned medium and the mRNA levels of HMGB1 receptors such as *TLR2*, *TLR4*, and receptor for advanced glycation end products (*RAGE*) in M1 and M2. Table 1 shows the HMGB1 concentrations in medium 3 h and 24 h after DIC treatment (500 µM). The HMGB1 concentrations in the medium of HepG2/M1 co-cultures after 3 h were significantly higher compared with those of HepG2 monocultures and HepG2/M2 co-cultures. The HMGB1 concentration in media of HepG2 monocultures and HepG2/M2 co-cultures were extremely low, although levels in the concentrated media could exceed the detection limits. Figure 6 shows the relative mRNA levels of *TLR2*, *TLR4*, and *RAGE* in M1 and M2 24 h after DIC treatment (500 µM). At 3 h after DIC treatment, there were only slight differences in *TLR2*, *TLR4*, and *RAGE* mRNA levels between M1 and M2. At 24 h after DIC treatment, the mRNA levels of *TLR2* in M2 were significantly decreased compared with those in M1. *TLR4* mRNA levels were decreased following co-culture with both M1 and M2 for 24 h.

## 3. Discussion

**Figure 7:**
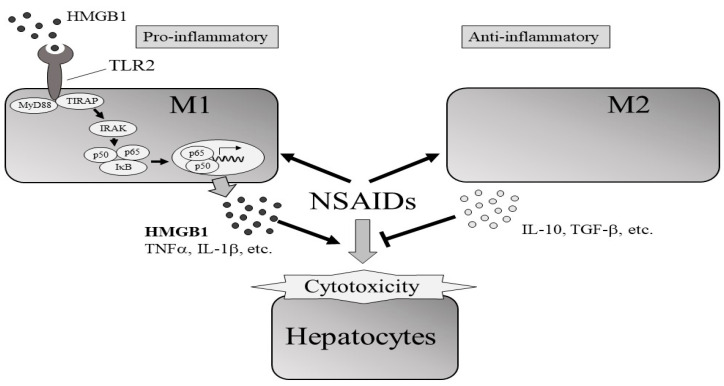
Effects of the presence of M1 or M2 macrophages on NSAIDs-induced cytotoxicity of hepatocytes. The protective effects of M2 on cytotoxicity could be partially explained by decreased signaling through the HMGB1/TLR2 axis in M2 compared with in to M1.

This study revealed the roles of M1 and M2 macrophages in DIC-induced cytotoxicity in HepG2 cells. Following DIC treatment and direct or indirect co-culture of M2 macrophages with HepG2 cells, cytotoxicity was reduced compared with HepG2 alone and HepG2/M0 and HepG2/M1 co-cultures. The decreased levels of HMGB1 in the medium of HepG2/M2 co-cultures 3 h after DIC treatment and *TLR2* expression in M2 24 h after DIC treatment could be partly involved in suppressing DIC-induced cytotoxicity (Figure 7).

Regarding the effects of co-culturing M2 with HepG2 cells on DIC-induced cytotoxicity, the ratio of M2 to HepG2 cells was important for the protective effect (Figure 2A). Ratios of HepG2 cells to M2 of 1:0.4 and 1:1 showed significantly decreased LDH release compared with 1:0. A sufficient accumulation of M2 to an inflamed site would likely allow cells to survive because the 1:0.4 ratio reflects the inflamed condition [17]. The 1:0.1 ratio reflected the physiological condition [17] and showed little effects on LDH leakage, suggesting that the accumulation of M2 to hepatocytes compared with the physiological condition would be important to increase the protective effects of M2 on DIC-induced cytotoxicity. It was also confirmed that the protective effect of HepG2/M2 (1:1) on DIC-induced cytotoxicity was enhanced, although it was higher than the ratio of macrophages in the inflamed liver. Co-cultures of M2 with HepG2 cells at 1:0.4 also showed significantly decreased NSAID-induced cytotoxicity from agents other than DIC except for ZOM (Figure 3), indicating that the inhibitory effects of M2 on cytotoxicity are not restricted to DIC. ZOM has been withdrawn from the market due to the higher cytotoxicity of reactive metabolites of ZOM [19,20]. These co-culture conditions with M2 and HepG2 cells might be insufficient to exert protective effects against ZOM-induced cytotoxicity. The promotion of M2 polarization in an inflamed site may help protect from drug-induced cytotoxicity. Approaches that change the type of macrophages predominantly in M2 and methods that accumulate M2 macrophages in the liver can be effective strategies for suppressing DILI. In addition, the co-culture system of HepG2 with M1 or M2 may be useful as a diagnostic tool in DILI. Both direct and indirect co-culture of M2 with HepG2 cells led to decreased DIC-induced cytotoxicity (Figure 2), suggesting that humoral factors secreted from M2–and not direct intercellular contact–transmitted the protective effect from M2 against DIC-induced cytotoxicity. Anti-inflammatory cytokines such as transforming growth factor-β and IL-10 are secreted from M2 [21,22]. These anti-inflammatory cytokines may exert a protective effect on DIC-induced cytotoxicity. The differences in the inhibitory effects on cytotoxicity between M1 and M2 were not explained by changes in *Bax* or *Bcl-2* mRNA levels (Figure 4), suggesting similar effects of the presence of M1 and M2 on the apoptosis pathway.

Zhang et al. demonstrated that HMGB1 can act as a trigger for non-apoptotic cell death [23]. Therefore, among the humoral factors, we focused on HMGB1 and HMGB1 receptors such as *TLR2, TLR4* [24,25], and RAGE. It has also been reported that HMGB1 facilitates M1 polarization [26]. HMGB1 concentrations in the media of HepG2/M1 co-cultures were significantly higher compared with those of HepG2 monocultures or HepG2/M2 co-cultures (Table 1). Among the HMGB1 receptors, *TLR2* mRNA levels were significantly decreased in M2 24 h after DIC treatment, while *TLR2* mRNA levels in M1 were unchanged 3 h after DIC treatment (Figure 6). These results suggested that the HMGB1/TLR2 signaling pathway could be suppressed in M2 but not in M1, leading to the different roles of M1 and M2 in NSAID-induced cytotoxicity. However, Teschke et al. reviewed diagnostic biomarkers of idiosyncratic liver injury retracted by EMA. HMGB1 is a sensitive biomarker for necrotic cell death, but not DILI specific. Therefore, it should be noted that the increased HMGB1 in HepG2/M1 compared with HepG2 and HepG2/M2 is not always an indicator of DILI [27].

*UGT1A9* mRNA levels were decreased and *MRP2* mRNA levels were increased in HepG2/M1 co-cultures (Figure 5). We hypothesize that these changes are a defensive response of cells, i.e., inhibition of NSAIDs-AG formation and promotion of NSAIDs-AG efflux, although changes in mRNA levels are not necessarily correlated with protein levels. Mitochondrial GSH transporters such as SLC25A39 and SLC25A40 [28] were unchanged between M1 and M2, suggesting that the distinct roles of M1 and M2 in NSAID-induced cytotoxicity could not be explained by differences in GSH transport to mitochondria. A limitation of our study is that some characteristics of HepG2 cells differ significantly from the normal biological condition due to the relatively low expression levels of CYP, UGT, and transporters in HepG2 cells. In particular, HepG2 cells exert the relatively low activity of CYP. In contrast to phase I enzyme activities, HepG2 cells maintain the phase II enzyme activities such as UGT [29]. Therefore, HepG2 cells are frequently used for toxicological studies but not drug metabolism studies. A comprehensive assessment of these protective effects on cytotoxicity that includes oxidative metabolites remains to be performed. Further studies using primary human hepatocytes and donor-specific monocytes macrophages are needed to clarify the detailed roles of M2 in DILI.

## 4. Materials and Methods

### 4.1. Chemicals and Reagents

Dulbecco’s modified Eagle’s medium (DMEM), Sepasol-RNA I Super G, and PMA were purchased from Nacalai Tesque, Inc. (Kyoto, Japan). IFN-γ, IL-4, and IL-13 were obtained from R&D systems, Inc. (Minneapolis, MN, USA). DIC, IBP, NAP, ZOM, and LPS from *Escherichia coli* were purchased from Sigma-Aldrich (St. Louis, MO, USA). ReverTra Ace was purchased from Toyobo Co., Ltd. (Osaka, Japan). The fast SYBR Green Master Mix and BCA protein assay kit were from Thermo Fisher Scientific (Waltham, MA, USA). Random primers, dNTPs, and LDH Cytotoxicity Detection Kit were purchased from Takara Holdings Inc. (Shiga, Japan). FLU, KET, RPMI-1640, and MS-grade porcine pancreatic trypsin were obtained from Fujifilm Wako Pure Chemical Co., Ltd. (Osaka, Japan). IBF was from Cayman Chemical Co. (Ann Arbor, MI, USA). The (glutathione disulfide) GSSG/GSH Quantification Kit was obtained from Dojindo Laboratories Co., Ltd. (Kumamoto, Japan). The Human HMGB1 ELISA kit was obtained from Arigo Biolaboratories Co. (Hsinchu City, Taiwan). Oligonucleotide primers were from Eurofins genomics, Inc. (Luxembourg, Luxembourg). All other chemicals and solvents were of MS grade or the highest commercially available purity.

### 4.2. Cell Culture

Human liver cancer HepG2 cells and human monocytic leukemia THP-1 cells (obtained from RIKEN BRC, Ibaraki, Japan) were maintained in DMEM and RPMI-1640, respectively, supplemented with 10% fetal bovine serum, 100 U/mL penicillin, and 100 µg/mL streptomycin. Cells were grown at 37 °C in a humidified incubator equilibrated with 5% CO_2_ and sub-cultured every 3 to 4 days. HepG2 and THP-1 cells were used with passages below 50. HepG2 cells were detached using 0.25% trypsin and 1 mm EDTA.

THP-1 cells were differentiated into M0 by treatment with 150 nM PMA (phorbol 12-myristate 13-acetate) for 24 h, and then culture in fresh RPMI-1640 for 24 h. M0 were differentiated into M1 by treatment with 10 pg/mL LPS and 20 ng/mL IFN-γ for 24 h. M0 were differentiated into M2 with 20 ng/mL IL-4 and 20 ng/mL IL-13 for 24 h. The differentiation of M0 to M1 or M2 was confirmed by the upregulation of IL-1β for M1 and of fibronectin for M2. HepG2 cells (1 × 10^5^ cells/cm^2^) were co-cultured with M0, M1, or M2 (HepG2/M0, HepG2/M1, or HepG2/M2) in fresh DMEM at ratios of HepG2 cells to M0/M1/M2 of 1:0, 1:0.1, 1:0.4, and 1:1. The HepG2 co-cultures were performed under direct (24-well plate, Corning, Inc., Corning, NY, USA) or indirect (24-well plate with Falcon cell culture inserts, pore size: 0.4 µm; upper compartment: M0/M1/M2; lower compartment: HepG2 cells) settings.

### 4.3. LDH Leakage Assay

HepG2, M0, M1, or M2 alone at 1 × 10^5^ cells/cm^2^ were treated with DIC at doses of 100, 200, 300, 500, 800, and 1000 µM. After 24 h, the LDH released into the medium was measured with the LDH Cytotoxicity Detection Kit [6]. LDH release in direct co-cultures of HepG2 cells with M0, M1, and M2 (1:0, 1:0.1, 1:0.4, and 1:1) or in indirect co-cultures (1:0 and 1:0.4) was measured 24 h after DIC treatments (500 µM). LDH release was measured in HepG2 cells or in indirect co-cultures of HepG2/M2 (1:0.4) 24 h after treatment with FLU (500 µM), IBF (1 mM), IBP (1 mM), KET (1 mM), NAP (1 mM), and ZOM (1 mM). These doses of the NSAIDs were set after confirming LDH leakage following NSAIDs treatment. Absorbance at 492 nm was measured using an absorption spectrometer (Sunrise R, TECAN Group Ltd., Mannedorf, Switzerland).

### 4.4. mRNA Levels of UGTs, Transporters, HMGB1 Receptors, and ER Stress-Associated Factors

Total RNA was extracted from HepG2 cells, M0, M1, and M2 3 h and 24 h after DIC or vehicle treatment using Sepasol RNA I Super G, and then reverse-transcribed into complementary DNA using ReverTra Ace qPCR RT Master Mix. Time points of 3 h and 24 h after DIC treatment were chosen because the relatively high concentrations of DIC-AG were detected 3 h after DIC treatment and the protein changes and cytotoxicity were observed 24 h after DIC treatment in the preliminary experiments and report [6]. The PCR mixtures were incubated at 95 °C for 10 s, and then amplified for 40 cycles of 95 °C for 5 s, 59 °C for 20 s, and 72 °C for 40 s using the Fast SYBR Green Master Mix [30]. The oligonucleotide sequences of the primers used for each mRNA target are shown in Table 2. Data were analyzed using the StepOne Real-Time PCR System (Thermo Fisher Scientific) and the multiplex comparative method. Target mRNA levels were normalized to those of β-actin.

### 4.5. Intracellular GSH Measurement

HepG2 cells alone or from indirect co-cultures with M1 or M2 (1:0.4) were treated with DIC (500 µM). After 3 h, intracellular GSH and GSSG levels were measured with the GSSG/GSH Quantification Kit. Absorbance at 405 nm was measured using an absorption spectrometer (Sunrise R) [31].

### 4.6. HMGB1 Concentrations in the Medium

HMGB1 concentrations in the medium of HepG2 cells alone or from co-cultures with M1 and M2 3 h and 24 h after treatment with DIC (500 µM) or vehicle were measured using the Human HMGB1 ELISA kit [32]. Briefly, 100 µL of medium was added to antibody-coated microplates. HRP-conjugated antibodies were added to each well. After incubation for 16 h at 4 °C, each well was washed with wash buffer. Next, 3,3′,5,5′-tetramethylbenzidine (TMB) substrate was added, and the absorbance was measured at 450 nm with an absorption spectrometer (Sunrise R). HMGB1 concentrations were estimated from a standard curve.

### 4.7. Statistical Analysis

Differences in between-group means were statistically analyzed using the Bonferroni test, or unpaired Student’s *t*-test. GraphPad Prism 5 (GraphPad Software, Inc., La Jolla, CA, USA) was used for all statistical analyses. Values of *p* < 0.05 were considered statistically significant.

## 5. Conclusions

This study showed that the presence of M2 macrophages decreases NSAID-induced cytotoxicity. The protective effects of M2 on cytotoxicity could be partially explained by decreased signaling through the HMGB1/TLR2 axis in M2 compared with in M1.

## Figures and Tables

**Table 1:** HMGB1 concentrations in the medium of HepG2, HepG2/M1, and HepG2/M2 3 h or 24 h after DIC treatment (500 μM).

	3 h	24 h
	HMGB1 (ng/mL)	
HepG2	ND	0.0371 ± 0.0371
HepG2/M1	1.78 ± 0.268 *** ^###^	0.201 ± 0.184
HepG2/M2	0.00719 ± 0.00719	0.294 ± 0.170

The results are expressed as the mean ± SE of each group (n = 4). Significant differences (*** *p* < 0.001 vs. HepG2 of 3 h, and ### *p* < 0.001 vs. HepG2/M2 of 3 h) were observed.

**Table 2:** Primer sequences used in RT-PCR assays.

Gene	Primer Sequence (5′–3′)	Product Size (bp)
*hBiP*	Forward: CTGGCTCCTAGAGTACAAGAAAAAG Reverse: ATATTGGTCTACAAGGAGCAGCAAC	177
*hCHOP*	Forward: ATTGCCTTTCTCTTCGGACAC Reverse: TTCTTCCTCTTCATTTCCAGGAGG	127
*hBax*	Forward: TCATGGGCTGGACATTGGAC Reverse: GCGTCCCAAAGTAGGAGAGG	145
*hBcl-2*	Forward: GAACTGGGGGAGGATTGTGG Reverse: GCCGGTTCAGGTACTCAGTC	125
*hUGT1A9*	Forward: GGGGGCATGAGGTGGTTGTA Reverse: CATTGAGCATGGGCAAAAGCT	142
*hUGT2B7*	Forward: ACAGCAACTGGAAAACAAGCA Reverse: ACACCAGCACCTTTCCACAA	121
*hMRP2*	Forward: AGTCACATGTCCATCCACTGTT Reverse: AGGATGACCTTTCATCCCAACC	83
*hSLC25A39*	Forward: TGCCCTTCTCAGCCCTGTA Reverse: CACAAAGCTCATGCCCACAG	101
*hSLC25A40*	Forward: ACCCACTCCCCAAAGGAAAATG Reverse: GTTTGTTGCCTCCCTCTTCAC	84
*hTLR2*	Forward: GACTCTACCAGATGCCTCCC Reverse: AAGTTATTGCCACCAGCTTCC	135
*hTLR4*	Forward: TGGATCAAGGACCAGAGGCA Reverse: GAGGACCGACACACCAATGA	141
*hRAGE*	Forward: CCCTGCTCATTGGGGTCATC Reverse: GTACTACTCTCGCCTGCCTC	139
*hβ-actin*	Forward: CACCATTGGCAATGAGCGGTTC Reverse: AGGTCTTTGCGGATGTCCACGT	135

## Data Availability

The datasets used and/or analyzed during the current study are available from the corresponding author on reasonable request.

## Abbreviations

AG	acyl glucuronide
Bax	Bcl-2 associated protein X
Bcl-2	B cell lymphoma/leukemia 2
DIC	Diclofenac
DILI	drug-induced liver injury
DMEM	Dulbecco’s modified Eagle’s medium
ER	endoplasmic reticulum
FLU	flurbiprofen
HMGB1	high mobility group box 1
IBF	ibufenac
IBP	ibuprofen
IFN	interferon
IL	interleukin
KET	ketoprofen
LDH	lactate dehydrogenase
LPS	lipopolysaccharide
MRP	multidrug resistance-associated protein
NAP	naproxen
NSAIDs	non-steroidal anti-inflammatory drugs
PMA	phorbol 12-myristate 13-acetate
RAGE	receptor for advanced glycation end products
TLR	toll-like receptor
UGT	UDP-glucuronosyl transferase
ZOM	zomepirac
